# Population Genomic Analysis of Ancient and Modern Genomes Yields New Insights into the Genetic Ancestry of the Tyrolean Iceman and the Genetic Structure of Europe

**DOI:** 10.1371/journal.pgen.1004353

**Published:** 2014-05-08

**Authors:** Martin Sikora, Meredith L. Carpenter, Andres Moreno-Estrada, Brenna M. Henn, Peter A. Underhill, Federico Sánchez-Quinto, Ilenia Zara, Maristella Pitzalis, Carlo Sidore, Fabio Busonero, Andrea Maschio, Andrea Angius, Chris Jones, Javier Mendoza-Revilla, Georgi Nekhrizov, Diana Dimitrova, Nikola Theodossiev, Timothy T. Harkins, Andreas Keller, Frank Maixner, Albert Zink, Goncalo Abecasis, Serena Sanna, Francesco Cucca, Carlos D. Bustamante

**Affiliations:** 1 Department of Genetics, Stanford University, Stanford, California, United States of America; 2 Institute of Evolutionary Biology (CSIC-UPF), PRBB, Barcelona, Spain; 3 CRS4 (Centre for Advanced Studies, Research and Development in Sardinia), Pula, Italy; 4 Istituto di Ricerca Genetica e Biomedica (IRGB), CNR, Monserrato, Italy; 5 Center for Statistical Genetics, University of Michigan, Ann Arbor, Michigan, United States of America; 6 Università degli Studi di Sassari, Dip. Scienze Biomediche, Sassari, Italy; 7 Laboratorios de Investigación y Desarrollo, Facultad de Ciencias y Filosofía, Universidad Peruana Cayetano Heredia, Lima, Peru; 8 National Archaeological Institute with Museum, Sofia, Bulgaria; 9 Sofia University St. Kliment Ohridski, Sofia, Bulgaria; 10 Life Technologies, Beverly, Massachusetts, United States of America; 11 University Hospital, Saarland University, Saarbrücken, Germany; 12 EURAC, Institute for mummies and the Iceman, Bolzano, Italy; University of California Santa Cruz, United States of America

## Abstract

Genome sequencing of the 5,300-year-old mummy of the Tyrolean Iceman, found in 1991 on a glacier near the border of Italy and Austria, has yielded new insights into his origin and relationship to modern European populations. A key finding of that study was an apparent recent common ancestry with individuals from Sardinia, based largely on the Y chromosome haplogroup and common autosomal SNP variation. Here, we compiled and analyzed genomic datasets from both modern and ancient Europeans, including genome sequence data from over 400 Sardinians and two ancient Thracians from Bulgaria, to investigate this result in greater detail and determine its implications for the genetic structure of Neolithic Europe. Using whole-genome sequencing data, we confirm that the Iceman is, indeed, most closely related to Sardinians. Furthermore, we show that this relationship extends to other individuals from cultural contexts associated with the spread of agriculture during the Neolithic transition, in contrast to individuals from a hunter-gatherer context. We hypothesize that this genetic affinity of ancient samples from different parts of Europe with Sardinians represents a common genetic component that was geographically widespread across Europe during the Neolithic, likely related to migrations and population expansions associated with the spread of agriculture.

## Author Summary

The analysis of the genome of the Tyrolean Iceman, a 5,300 year old mummy from Central Europe, revealed a surprising recent common ancestry with modern Sardinians for this ancient genome. However, this study was limited both by the availability of data from Sardinians and by a lack of genomic data from other ancient European samples. Here, we use genomic data from modern Sardinians and from ancient European individuals from different geographic regions and cultural contexts, to demonstrate that this ancestry component is shared among individuals associated with the onset of agriculture in Europe. Our results thus suggest that the Iceman's Sardinian ancestry actually reflects a more widespread genetic component related to the migration of people during the Neolithic transition in Central Europe.

## Introduction

The Tyrolean Iceman is a well-preserved natural mummy that was discovered on a glacier near the Austrian-Italian border in 1991. Subsequent analyses revealed that the remains were from a ∼45-year-old male, with a ^14^C dating estimated age of 5,300 years, making it the oldest human mummy discovered in Europe to date [1]–[3]. This spectacular discovery provided a unique view of the cultural and socio-economic context of the Central European Alpine region during the late Neolithic and Copper Age (e.g. [4]). More recently, the publication of the Iceman's nuclear genome sequence allowed us to address the question of his origin from a genomic perspective, revealing a surprising genetic affinity with individuals from Sardinia [5]. The Iceman's Y-chromosome lineage was determined to be G2a-L91, which is found at appreciable frequencies only in Corsica and Sardinia but is rare elsewhere in Europe [5]. Furthermore, in a principal component analysis including more than 1,300 Europeans, the Iceman clustered outside of the genetic variation of the continental Europeans and close to individuals from Sardinia. However, although a previous study on bone and tooth stable isotope compositions concluded that the location of the Iceman's origin could be restricted to a few valleys close to the discovery site [6], the question of whether this signal reflects an ancestry component more widespread in Europe during the Neolithic could not be answered from the analysis of a single individual.

Most studies of the genetic structure of prehistoric European populations to date have described patterns of variation in the mitochondrial DNA (mtDNA), primarily due to the limitations of sequencing the nuclear genome from ancient samples with low endogenous DNA contents. Nevertheless, the limitation of only investigating the maternal lineage has been offset by the capacity to investigate larger sample sizes, both spatially and temporally distributed, which has yielded considerable insight into the genetic history of Europeans [7]–[12]. More recently, and subsequent to the publication of the Iceman's genome, two additional studies of ancient Europeans have expanded the view to include autosomal loci [13], [14]. The publication of these autosomal datasets, together with recently available whole-genome sequencing datasets from modern Europeans, therefore allows a re-examination of the Iceman's ancestry together with these new data. In particular, under the hypothesis that this Sardinian-like ancestry component was more widespread in Neolithic Europe, we would expect that individuals from a similar time period as the Iceman but a different geographic origin would show a comparable pattern of relatedness to Sardinians. To address this hypothesis, we compiled and analyzed datasets from modern and ancient Europeans, including previously unpublished whole genome sequencing data from 452 Sardinians, as well as ancient DNA data from two 2,500-year-old ancient Thracian individuals from Bulgaria, which we will refer to as Thracians. These datasets allow us to address the question of the Iceman's ancestry and its implications with respect to the genetic structure of Neolithic Europe.

## Results

### Datasets

**Figure 1:**
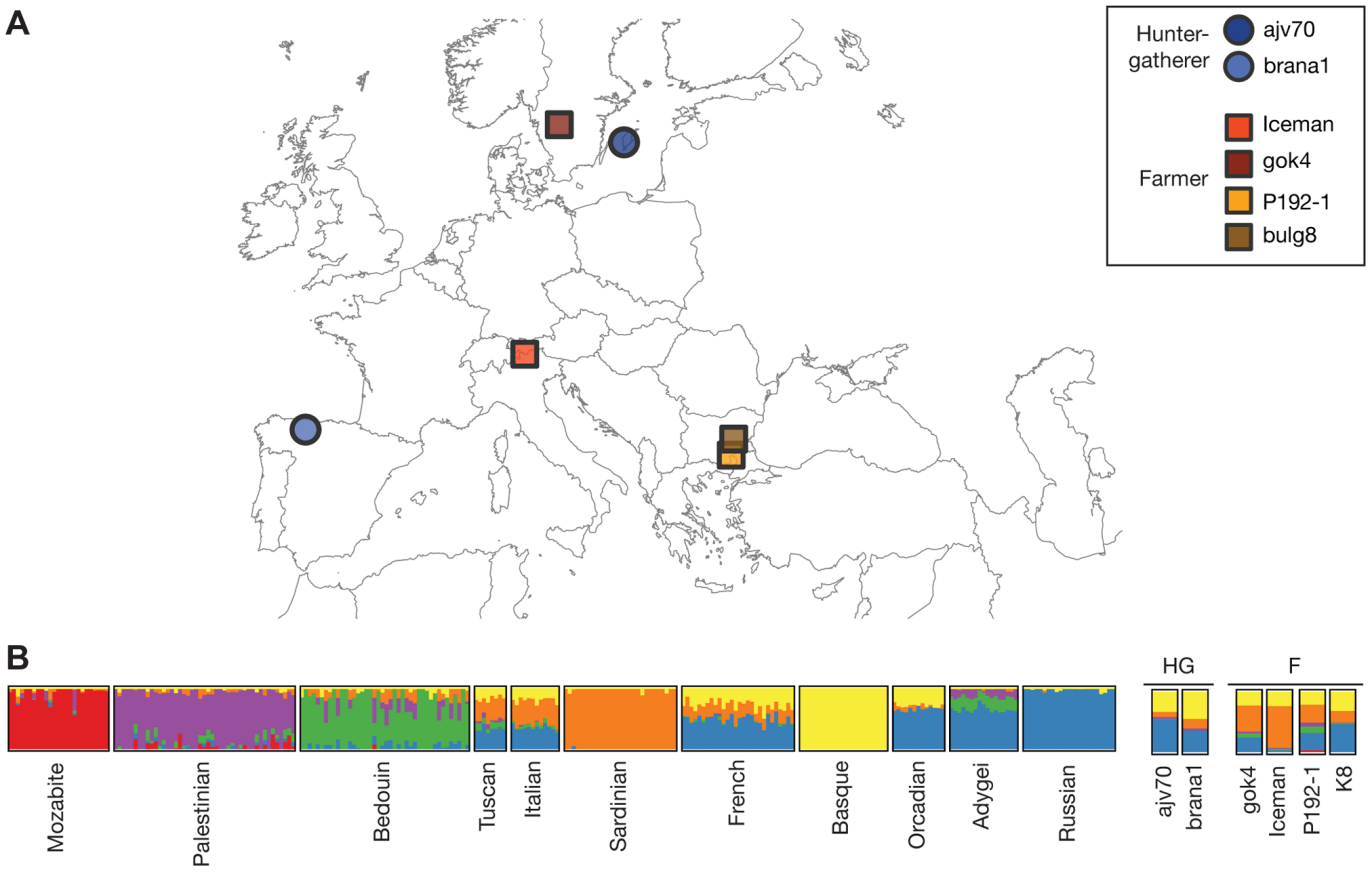
Geographic origin of ancient samples and ADMIXTURE results. (A) Map of Europe indicating the discovery sites for each of the ancient samples used in this study. (B) Ancestral population clusters inferred using ADMIXTURE on the HGDP dataset, for *k* = 6 ancestral clusters. The width of the bars of the ancient samples was increased to aid visualization.

We compiled several sets of single nucleotide polymorphism (SNP) genotype data for our analysis. For each dataset, data from the ancient genomes was merged into a reference set of modern populations, using only SNPs present in the modern data. Three complementary reference datasets were used: SNP array genotype data from the Human Genome Diversity Panel (HGDP); high coverage whole-genome sequencing data from Complete Genomics (CG); and low coverage whole-genome sequencing data from the 1000 Genomes project and Sardinians (1000G/Sardinia). For the 1000G/Sardinia dataset only population-level allele frequency data was used, to circumvent lower genotype accuracy in the low-coverage experimental design. Although the CG dataset does not include individuals from Sardinia, we included it to investigate the relationship of the ancient individuals with Eurasians using high-coverage whole-genome data. The ancient genomes we used comprised both previously published and newly generated data. In addition to the Iceman genome, we included partial genomic DNA data from five individuals distributed across Europe (Figure 1A). We broadly classified all ancient samples into hunter-gatherers (HG) or farmers (F), based on the context of their respective cultural attributes. The HG group included a 7,000-year-old Mesolithic individual (*brana1*) from the Iberian Peninsula [14], as well as a Neolithic Swedish individual (*ajv70*) from Sweden [13], both previously published. Both of these studies included data from more than one HG individual in their analyses. However, given that the results in both studies suggested comparable ancestry for the HG samples, we focused only on the individual with the highest coverage for our analysis. The remaining four individuals were classified as farmers. The F group included the Iceman, a Neolithic Swedish farmer (*gok4*) from the same study as the Swedish hunter-gatherer [13], and new DNA sequencing data from two ancient Thracian Iron Age individuals from Bulgaria (*P192-1*, *K8*) [15]. A summary of the final datasets is shown in Table 1.

**Table 1:** Summary of datasets.

Sample	Region	Age [Years]	Group*	Number of SNPs in final merge		
				HGDP†	CG	1000G/Sardinia
Iceman	Austria/Italy	5,300	F	162,537	3,730,194	7,974,125
*ajv70*	Sweden	5,300–4,400	HG	12,931	387,642	730,201
*gok4*		4,921±50	F	7,250	220,491	434,348
*brana1*	Spain	6,980±50	HG	3,996	123,938	225,271
*P192-1*	Bulgaria	2,800–2,500	F	1,270	40,022	75,510
*K8*		2,450–2,400	F	1,046	32,767	59,128

*HG: Hunter-gatherer; F: Farmer.

† only SNPs ascertained in non-European populations.

### Iceman within contemporary Europe

We used the unsupervised clustering algorithm ADMIXTURE [16] to investigate the relationship of the Iceman with 11 European, Middle Eastern and North African populations in HGDP, using only SNPs discovered in non-European populations to avoid biases from differential relatedness of European populations to the discovery populations. Ancestral clusters were inferred for the modern samples only, with the number of ancestral clusters *k* ranging from *k* = 2 to *k* = 8 (Figure S1). We then determined the most likely cluster proportions of the Iceman using the inferred ancestral cluster allele frequencies from the modern samples. At smaller *k* values, estimated ancestry proportions of the Iceman match those of other individuals from Southern Europe, including a small estimated proportion of Middle East-related ancestry (Figure S1). For higher values of *k*, the majority of the Iceman's ancestry is composed of the Sardinian-related cluster, with a small proportion of the Basque-related cluster (Figure 1B). Principal component analysis (PCA) recapitulates this picture, with the Iceman clustering close to the Sardinian individuals, although somewhat shifted towards the Northern Italian individuals (Figure S2).

**Figure 2:**
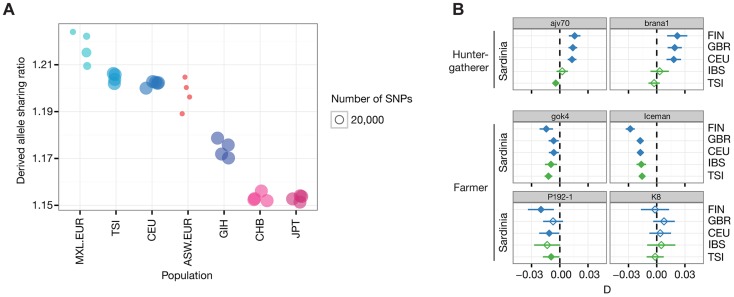
Allele sharing and D-tests with whole-genome datasets. (A) Normalized derived allele sharing rate of the Iceman with Eurasian whole genomes from Complete Genomics. Each circle represents the rate of sharing with a particular genome, grouped by population of origin. Positions on the y-axis have added jitter for ease of visualization. Populations with EUR suffix correspond to the European ancestry tracts of individuals of populations with known European admixture (ASW, MXL). Due to differences in admixture proportions among individuals from those populations, the total number of observations varies between individuals, indicated by the size of the circles. (B) D-test results for the ancient samples compared to populations from the 1000 Genomes project and Sardinia. Each panel shows results for a particular ancient sample, grouped by cultural context. Diamonds indicates the value of the D-statistic for a single D-test involving the ancient sample and a pair of modern populations, shown on the left and right of the panels. Significance at Z = 3 is indicated with filled diamonds, and the line shows the corresponding standard error of the D-statistic. Plot colors indicate different pairs of geographic regions within Europe (blue: Europe S/Europe N; green: Europe S/Europe S).

To determine the relationship of the Iceman with the CG genomes, we quantified the rate of derived allele sharing with each of the modern samples (Figure 2A, Figure S3). The highest sharing was found with European genomes (TSI, CEU), consistent with the European origin of the Iceman. However, within Europe we did not observe a noticeable difference between the Southern European TSI and the Northwestern European CEU. To further investigate this result, we computed the D-statistics [17], [18] of the configuration D(Outgroup, Iceman; Population 1, Population 2), where populations 1 and 2 correspond to a pair of modern populations. When we test the configuration D(O, Iceman; TSI, CEU) we find no significant deviation from zero (D = −0.007, Z = −2.07), indicating that neither CEU nor TSI are more closely related to the Iceman (Figure S4). Interestingly, the European ancestry segments from the Mexican genomes (MXL) show the highest overall derived allele sharing within Europe (Figure 2A). Combining across all MXL European tracts, the D tests of the form D(O, Iceman; MXL.EUR, CEU/TSI) are both significantly different from zero (Z_CEU_ = −7.16, Z_TSI_ = −5.14) (Figure S4), indicating a closer relatedness of the Iceman with the MXL European segments than either CEU or TSI. One possible explanation for this observation would be that the source population for the European admixture in Mexicans was indeed more closely related to the Iceman than either the TSI or the CEU. However, we do not find a difference in relatedness between modern Iberians and either the TSI or CEU in the 1000G/Sardinia dataset described below. Gene flow from a more distantly related population into TSI or CEU after their split from the ancestral population of the MXL European segments would also cause a decrease in matching with the Iceman. A related explanation is that the excess sharing results from comparing only double European segments in the Mexican individuals to those of the whole genome in CEU and TSI. Since our local ancestry inference masks segments that are not clearly of European ancestry, any non-European ancestry in the CEU or TSI would lead to excess sharing of the European tracts in the MXL and the Iceman. For example, we and others have previously reported a gradient of North African ancestry within Europe and this may be a source of differential non-European ancestry among the CEU, TSI, and double European segments in the MXL. North African gene flow into Europe likely occurred after the death of the Iceman, so he would not carry many North African segments and thus would appear more closely related to the high-confidence European segments in the MXL. Another potential source of non-European ancestry in modern day Europeans is gene flow from other parts of Asia (e.g., the Finns appear to carry significant amounts of non-European Eurasian ancestry - see below).

**Figure 3:**
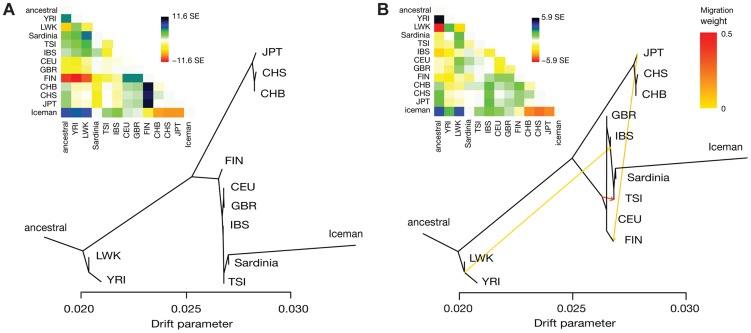
Results of TreeMix analysis of the Iceman with 1000G/Sardinia. Shown are maximum-likelihood trees and the matrices of pairwise residuals (inset) for a model allowing (A) *m* = 0 and (B) *m* = 3 mixture events. Large positive values in the residual matrix indicate a poor fit for the respective pair of populations. Edges representing mixture events are colored according to weight of the inferred edge.

We then investigated the relationship between the Iceman and all pairs of European populations in the 1000G/Sardinia dataset as described above. We find that all significant tests fall into one of two categories, involving either the Finns (FIN) or Sardinians (Table S1, Figure S5). In all tests involving the FIN the Iceman is significantly more closely related to the non-Finnish population, irrespective of which population is used. Furthermore, the Iceman is always significantly more closely related to Sardinians than any other European population (Figure 2B), consistent with a more recent shared ancestry with Sardinians. To further investigate this scenario, we modeled the relationship of the Iceman with the modern populations using *TreeMix* [19]. The maximum-likelihood tree without migration edges shows the expected relationships corresponding to the three continental groups included in this dataset (Figure 3A). Within the European clade, we find a North-to-South pattern of population splits starting with the FIN as outgroup to all other Europeans. Importantly, the Iceman consistently forms a clade with the Sardinians within the group of Southern Europeans (98% bootstrap), in agreement with the results of the D-test. Bootstrap support is high (>95%) for all other splits, with the exception of the edge grouping TSI with Sardinians and the Iceman, which is slightly less supported (89%). The long branch to the Iceman is likely a consequence of higher error rates and/or reduced heterozygosity in the ancient sample, mimicking a population exhibiting increased genetic drift and differentiation (e.g. see also [20]). Examination of the residuals (Figure 3A inset) indicates some population relationships that are not fully resolved by the simple tree model (e.g., FIN and Asian populations). Allowing for up to five migration edges on the tree, we recover mixture events consistent with previous results (Figure 3B; see below for discussion). Nevertheless, the clade Iceman/Sardinians remains strongly supported (>96% bootstrap) for all models irrespective of the number of mixture events included (Figure S6).

To gain further insight into the demographic history of the Iceman, we used a coalescent-based method [21] to estimate divergence times between the Iceman genome and the genomes of the individuals in the CG and 1000G/Sardinia datasets. We find that the estimated divergence times between the Iceman and the CG genomes are within the expected range for an individual with European ancestry, and in line with recent results based on the whole-genome sequencing data [22], [23] (Table S2). Within Europe, most individuals show estimates between 3,000 and 4,500 YBP, roughly corresponding to the age of the Iceman. In addition, a recent study on genetic ancestry in Europeans inferred from patterns of identity-by-descent across the genome also indicates a signal of demographic events in a shared ancestral population with a comparable age [24]. For the 1000G/Sardinia dataset, we estimated divergence times comparing the Iceman to population-representative genomes, constructed by randomly sampling alleles proportional to their frequency in each population. The divergence times we obtain are in line with the times estimated from the CG genomes, with higher estimates for Northern European populations, in particular the FIN (9,196 YBP; Table S3). In Southern Europe, both Sardinians and TSI show very low estimates (TSI 1,709 YBP; Sardinia 2,321 YBP). Surprisingly, we found that the divergence from Iberians (IBS) was higher than the FIN from Northern Europe (13,806 YBP; CI 1,957–29,739)). A possible explanation could be the presence of non-European ancestry in the IBS, through gene flow from North Africa as previously described [25] and discussed above.

### Relationship with other ancient genomes

The results described in the previous section strongly suggest that among contemporary European populations, Sardinians are most closely related to the Iceman. To investigate whether other ancient Europeans show a similar pattern, we compiled genomic data from five additional individuals of varying ages and cultural contexts and merged them with the Iceman datasets (Table 1). We first inferred ancestral clusters for these individuals using the HGDP ADMIXTURE methodology described above. We find that at low values of *k*, ancestry proportions are similar among the different ancient samples, and comparable to other European populations. We note that at *k* = 3, populations from Southern Europe show a small proportion of ancestry related to Middle Eastern populations, which is absent from the two hunter-gatherer (HG) individuals, but present in the farmer (F) individuals at a comparable level (Figure S1). At *k* = 6, the HG group can be distinguished from the farmers by a smaller proportion of the Sardinian component, which is also the dominant component in both the Iceman and the Swedish farmer (*gok4*) from the F group (Figure 1B). Within the F group, *gok4* has a slightly lower proportion of the Sardinian cluster than the Iceman, and most closely resembles the Tuscans and Northern Italians, in agreement with the results of Skoglund *et al.* [13]. Both of the HG individuals have previously been shown to cluster with Northern European populations (16, 17), and in concordance with these results we find the highest proportion of Northern European/Russian ancestry in those individuals. Interestingly, the Iberian HG (*brana1*), which was discovered geographically close to contemporary Basque populations, also shows the highest proportion of Basque ancestry. For the Thracian individuals from Bulgaria, no clear pattern emerges. While *P192-1* still shows the highest proportion of Sardinian ancestry, *K8* more resembles the HG individuals, with a high fraction of Russian ancestry. Because those two individuals also had the lowest number of SNPs, we wanted to investigate the accuracy of cluster assignments from the modern individuals. We inferred ancestry proportions for each modern individual for all SNP subsets corresponding to the ancient samples, and calculated the root mean squared error (RMSE) per population and *k* value compared to the full dataset. We find that the accuracy is very high for the Iceman (RMSE< = 0.01) and remains high for *gok4* and the HG samples (RMSE< = 0.05 for most clusters; Figure S7). Both ancient Thracians show RMSE values between 0.05 and 0.10, suggesting greater uncertainty in the estimates for those samples, also seen in the PCA results for those samples (Figure S2).

We then used the 1000G/Sardinia dataset to gain further insight into the relationship of the other ancient genomes with Sardinia. Using the D-test as described above, we observe a strong differentiation between HG and farmer individuals in their affinity with the modern European populations. We find that the HG individuals are always significantly closer to Northern European populations (FIN, CEU, GBR) (Figure 2, Figure S5), whereas the farmer individuals generally show a closer relationship with Sardinia than with the other European populations. For *gok4* the pattern is particularly striking, closely resembling the Iceman in its relationship with Sardinians. Results for *P192-1* also follow this pattern, although some comparisons fail to reach significance, likely because of reduced power due to a lower number of SNPs. However, results are inconclusive for the other Thracian individual (*K8*), where all but one comparisons are non-significant (Z< = 3) (Figure S5), again likely due to a smaller number of SNPs in this sample. Results from the *TreeMix* analysis for each ancient genome show a similar picture. In the HG group, the Iberian *brana1* forms an outgroup to all other European populations (100% bootstrap), whereas *ajv70* splits after the FIN but before other Northern Europeans (95% bootstrap) (Figure S8). In the F group, both *gok4* and *P192-1* form a clade with Sardinians like the Iceman, although with less bootstrap support (*gok4* 83%, *P192-1* 56%). Finally, although *K8* clusters with Northern European populations, its position in the tree is not resolved (3% Bootstrap). We note however that despite the reduced number of SNPs for *K8*, the relationships among the modern populations are consistent with the full dataset and generally well supported (bootstrap >90%), except within the Southern European group (minimum bootstrap 53%). It is therefore possible that the inconclusive pattern for *K8* either reflects a possible higher level of modern DNA contamination (see Table S4 in [15]) or a more complex relationship to the modern populations included in the analysis.

### Signals of admixture

To further investigate the history of the populations in the 1000G/Sardinia dataset, we investigated patterns of admixture. We first turned to the *TreeMix* analysis of the merged dataset with the Iceman described above, allowing up to five mixture events. The first four inferred edges are highly significant (p<10^−10^ for *m* = 1 to *m* = 4), the last one however to a much lesser extent (p = 0.009). In addition, both the amount of variance explained and the residuals do not change substantially after *m* = 4, indicating possible issues with overfitting the model at *m* = 5. We therefore report estimates of the tree with four inferred mixture events in the remainder of this section. The first inferred edge corresponds to sub-Saharan African admixture in Southern European populations (edge weight *w* = 0.027), which is consistent with previous estimates of 1%–3% sub-Saharan African ancestry in those populations via North African gene flow [25], [26]. We also infer that the FIN trace around 7% of their ancestry (*w* = 0.075) to present-day Japanese (JPT), consistent with evidence of circumpolar gene flow [27]. A higher similarity of Finns with Asians than other European populations has also been observed previously, in particular in Eastern Finns [28]. At *m* = 3 migrations, an edge is added between the ancestral Europeans and the TSI, with a surprisingly high proportion (*w* = 0.35) (Figure 3B), which is somewhat more difficult to interpret. The last edge added corresponds to a mixture of an Iceman-related population and the Bantu-speaking Luhya (LWK) from Eastern Africa (*w* = 0.03). The LWK have previously been reported as showing a signal of gene flow of possible Neolithic Middle Eastern or European origin [29], which would be consistent with the observed signal (see also [30], [31]).

We proceeded to investigate these signals of admixture in a less parameterized way, using *f_3_* statistics [18]. We calculated *f_3_* for all populations in the 1000G/Sardinia dataset as targets, using two sets of combinations of source populations. In the first set, we use all pairs of remaining modern populations as potential sources, whereas in the second analysis, we always use the ancient samples as one of the source populations. We find a large number of significant tests (Z≤−3), which we group based on interpretation (Table S4, S5). A large fraction of these reflect the migration edges inferred in the *TreeMix* analysis. We find evidence for admixture in the LWK using any of the non-African population as source populations (max Z = −9.7), which remains significant if we replace the non-Africans with either HG or F ancient samples (max Z = −3.4). The TSI show the signature of African admixture as described when using CEU, GBR or Sardinia as European source populations (max Z = −4.4), whereas for the IBS only YRI and GBR as source populations gives a significant result (Z = −3.4). We also confirm the Asian-related admixture in the FIN when using the other Northern European populations as one source (max Z = −5.8). The remainder of the significant results within Europe follows the pattern of a south-to-north gradient of genetic similarity, with Sardinians and Finns at either ends of the gradient (Sardinia – IBS/TSI – CEU/GBR – FIN), consistent with a model of isolation by distance as previously described [32]–[34], For example, all *f_3_* tests using Sardinians as one source population and TSI/IBS as the target population are significant irrespective of which Northern European population is used as the other source (Table S4), whereas for the CEU/GBR, all tests of the form *f_3_*(CEU/GBR;Europe S,FIN) are significant. A complementary explanation suggested by recent results would be a scenario of increasing levels of admixture between arriving farmers and local HG populations during the Neolithic transition [13], [18]. In the case of this second scenario, we would predict that replacing one modern European source population with its corresponding ancient sample of HG or farmer origin should show comparable results in the *f_3_* test. This is indeed what we observe in the majority of the cases. For example, tests using either TSI or IBS as target population remain largely significant if we replace Sardinians with the Iceman as one of the source populations. In addition, in tests with CEU or GBR as target populations, the HG samples *ajv70* and *brana1* can replace the FIN, whereas the Iceman and *P192-1* from the farmer groups can replace the southern source populations (Table S5). However, while the strongest signal using the modern populations is observed with the Sardinians as southern source (e.g. *f_3_*(CEU;Sardinia,FIN), Z = −25.4), tests using the HG samples only reach significance with the TSI as the second source population. Taken together, these results suggest that the observed patterns can at least in part be explained by different levels of HG ancestry proportion in Northern versus Southern Europe, as previously suggested [13], [18].

The HGDP dataset also contains genotype data for Neanderthal, Denisova, and Primate samples in addition to modern humans. We therefore used this dataset to perform PCA as previously described [35]. Specifically, we first built the PCA space using only data from Denisova, Neanderthal and Chimpanzee, and we then projected all modern individuals as well as the Iceman onto the first two inferred principal components. In agreement with previously reported results, modern human samples separate into three distinct clusters (Figure S9). We see that all non-African populations are shifted towards the Neanderthal in PC space, whereas populations from Oceania form their own cluster with an additional shift towards the Denisovan. The Iceman clearly falls within the cluster of the non-African populations (except Oceania), indicating a shared signal of Neanderthal admixture with other European and Asian samples. To confirm this signal, we merged data from both the Neanderthal and the Denisovan genome with the CG dataset and carried out D-tests as described above. In particular, we calculated all D-statistics of the form D(O,archaic;Iceman,modern), where ‘archaic’ represents either Neanderthal or Denisova and ‘modern’ represents all modern populations in the CG dataset (Figure S10). To eliminate potential confounding due to shared DNA damage patterns between the Iceman and the archaic hominins, we excluded all transition sites for this analysis. We find that both Neanderthal and Denisovan are significantly more closely related to the Iceman than to African populations, consistent with previously reported results for modern non-Africans [36]. However, all D-tests involving another non-African population do not significantly deviate from zero, suggesting that the Iceman genome contains levels of archaic ancestry that are comparable to that of other non-African populations.

## Discussion

The discovery of a genetic affinity between the Iceman and modern-day Sardinians showcased both the power and some of the remaining limitations of the availability of nuclear genomic data from historical human samples [5]. While the result was unexpected with respect to the known history and geographic location of the individual at the time, the broader interpretation of the finding was hindered by the question of how representative this single individual really was for the Central Alpine region. Partial genomic sequences from ancient individuals published after the Iceman's genome have shed additional light onto the genetic structure of Neolithic Europe, in particular with respect to the relationship between the hunter-gatherer populations descending from the first Europeans and individuals associated with the spread of farming during the Neolithic transition [7], [8], [13], [14]. Here, we compiled large-scale genomic datasets, including both recently published and newly generated data from ancient Europeans, to reexamine the evidence for the Iceman's relationship to Sardinia and to address the question of the broader historical context of this relationship. Our results using both the HGDP array data and whole-genome sequencing data confirm the Iceman's genetic relationship with Sardinian populations. For example, in the 1000G/Sardinia dataset, which we expect to provide the highest statistical power due to the large number of markers, we found that the Iceman consistently forms a clade with Sardinians, irrespective of the number of migration edges we allow in the model. The less parameterized D-test results also confirm this, with all pairwise comparisons involving Sardinians being highly significant (Figure 2; −10.2≤Z≤−17.7).

The results of the analyses including additional ancient genomes provide mounting evidence that the Iceman's genetic affinity with Sardinians reflects an ancestry component that was widespread in Europe during the Neolithic. Despite their different geographic origins, both the Swedish farmer *gok4* and the Thracian *P192-1* closely resemble the Iceman in their relationship with Sardinians, making it unlikely that all three individuals were recent migrants from Sardinia. Furthermore, *P192-1* is an Iron Age individual from well after the arrival of the first farmers in Southeastern Europe (more than 2,000 years after the Iceman and *gok4*), perhaps indicating genetic continuity with the early farmers in this region. The only non-HG individual not following this pattern is *K8* from Bulgaria. Interestingly, this individual was excavated from an aristocratic inhumation burial containing rich grave goods, indicating a high social standing, as opposed to the other individual, who was found in a pit [15]. However, the DNA damage pattern of this individual does not appear to be typical of ancient samples (Table S4 in [15]), indicating a potentially higher level of modern DNA contamination. On the other hand, the Swedish and the Iberian hunter-gatherers show congruent patterns of relatedness to the modern populations of Northern Europe, which is consistent with the previous results using those samples.

Sardinians have long been recognized as forming a distinct outlier within contemporary European genetic diversity (e.g. [37]), often interpreted as a consequence of genetic isolation and/or founder effects in the demographic history of the island. It is thought that permanent settlement of the island was established by around 10,000 YBP, and recent results from both genetic and cranial morphological data suggest population continuity since the Neolithic [38]–[40]. Our results support this continuity and indicate that gene flow from mainland Europe during the time of the spread of agriculture in Europe contributed significantly to the present Sardinian gene pool. It is important to point out that this does not imply that early Palaeo-Mesolithic settlers did not contribute to the genetic diversity observed in Sardinia today. In fact, D'Amore *et al.* argue for a substantial contribution of Mesolithic settlers, but also find evidence for a later contribution of people from the mainland during the Bronze and early Copper Ages, which is compatible with the age of the farmer individuals analyzed here [38]. We therefore hypothesize that Sardinia, by remaining largely isolated from the later events that shaped genetic variation in mainland Europe, provides a modern-day “snapshot” of the genetic structure of the people associated with the spread of agriculture in Europe (see also [18]).

**Figure 4:**
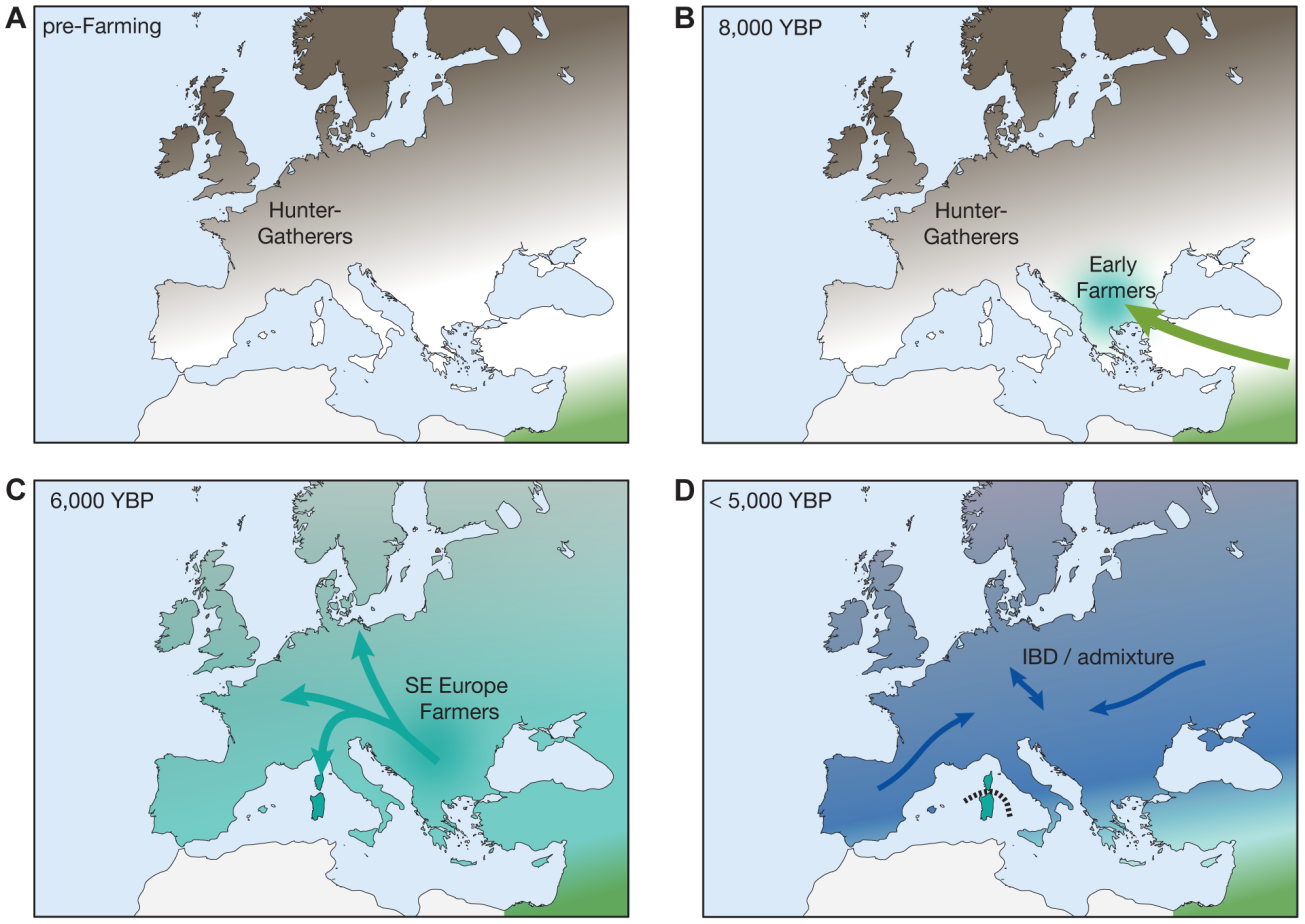
A simplified model for recent demographic history of Europeans. The panels indicate a possible demographic scenario consistent with the observed signals. (A) Mesolithic HGs present in mainland Europe prior to the arrival of agriculture. (B) Initial spread of farming from the Middle East beginning from 7,000 YBP into SE Europe. (C) Expansion of farming to N Europe from SE European gene pool and establishment of main S-N gradient of genetic diversity. Wave of migration also reaches Sardinia. (D) Continuous population expansion and admixture with local HGs as well as additional migrations continues to shape genetic diversity in mainland Europe, but Sardinia remains mostly isolated (IBD: isolation by distance).

Building on the results of previous studies, we outline a simplified scenario for the demographic history of Europe during the Neolithic in Figure 4. It is important to note that this illustration is a simplification and certainly not adequate to explain the full complexities of European demographic history. For instance, a recent study using mtDNA data from 364 prehistoric Europeans suggests a number of marked shifts in the genetic makeup of Neolithic Central Europeans, particularly during the Middle and Late Neolithic stages (<6,000 YBP) [41]. It nevertheless provides a reasonable outline consistent with the broad patterns of ancestry presented in this and other recent studies, in particular with respect to the observed Sardinian affinity of the early farmers. In agreement with previous studies, we find that both HG individuals closely resemble each other in their relationships with modern Europeans despite considerable geographic distance, suggesting relative homogeneity in the HG gene pool prior to the spread of farming in Northern and Western Europe (Figure 4A) [7], [42]. Furthermore, there is a large body of evidence (reviewed in [42]) suggesting a genetic contribution of early Neolithic farmers originating in the Middle East during the initial spread of agriculture into Southeastern Europe. While we do find that a small proportion of a Middle Eastern ancestry component distinguishes the farmer individuals from the HG in the ADMIXTURE analysis (Figure 1A; Figure S1, *k* = 3), they generally resemble Southern European populations rather than Middle Eastern ones. A possible explanation for this observation could be that there was an initial wave of migration from the Middle East into Southeastern Europe during the early Neolithic transition, followed by a subsequent expansion of a derived Southern European population with some fraction of Middle Eastern ancestry towards Central and Northern/Western Europe (Figure 1B and 1C). The secondary expansion would include substantial migration to Sardinia, leading to the observed genetic affinity of the ancient farmers descending from these migrants to present-day Sardinians. Interestingly, a recent study of mitochondrial genomes from 39 ancient individuals from Central Europe [11] suggests that early Neolithic mtDNA lineages in Central Europe introduced by the first farmers from the Middle East were largely replaced by events during the Middle Neolithic starting around 4000 BC, which would be consistent with this scenario. Differential levels of admixture of the expanding farmers with local hunter-gatherer populations then establish the main pattern of genetic diversity observed in Europe today, together with additional migration events (such as the ones suggested in [41] as mentioned above), but excluding Sardinians due to their subsequent isolation (dashed line, Figure 4D). For example, one such later migration event would be the expansion of the Bell Beaker culture described in Brotherton *et al.* [11], where we also find that the IBS show a closer relationship with the CEU/GBR than the TSI (Figure 3). On the other hand, a recent preprint analyzing newly sequenced ancient European genomes suggests three ancestral populations for modern Europeans [43], a hypothesis that was not addressed in our study given the limitation of the ancient samples available. Nevertheless, the main results in that study are largely consistent with the results presented here. For example, the authors suggest that early farmers from Central Europe already show evidence of admixture with European hunter-gatherers, and that Sardinians derive ∼90% of their ancestry from this early farmer population, consistent with our basic model described above [43].

Finally, an important caveat with analyses based on allele sharing such as the D-test is their sensitivity to confounding factors such as correlated errors in a pair of samples used in the test [44]. This is particularly a problem when using one ancient sample as an ingroup in the D-test, since correlated DNA damage patterns, or differences in sequence quality between the ancient samples, can lead to an over- or underestimation of allele sharing between them, respectively (e.g., also discussed in [44]). The exclusion of all transition sites from the analysis is commonly used to circumvent this problem; however, other more subtle biases shared among the ancient samples cannot be ruled out. For example, after excluding all transition sites, we find that the test D(O,Iceman;*gok4*,Sardinia) is significantly negative (D = −0.05, Z = −3.0), indicating that the Iceman is more closely related to the Swedish farmer. However, the test D(O,Iceman;*brana1*,Sardinia) shows an even stronger signal in the same direction (D = −0.09, Z = −3.5), which would suggest that the Iceman is also more closely related to the Iberian hunter-gatherer than to Sardinians. Given the observed discontinuity in the relationship of hunter-gatherers and farmers with modern Europeans, we believe that this pattern is at least in part due to unaccounted biases as described above. We therefore did not analyze the relationship of more than one ancient sample combined with modern samples using D-tests throughout the study. The aforementioned issues also highlight the need for improved statistical methods that will allow the analysis of ancient and modern samples in joint fashion, while at the same time accounting for differences in data quality and biases due to DNA damage and differences in sequencing technology. The incorporation of these methods with additional genomic and archaeological data from key spatial and temporal points will be crucial in the quest to disentangle the complex population history of the European continent.

## Materials and Methods

### Sequencing of Thracian individuals

The two individuals from Bulgaria used in this study originate from two separate excavations, both associated with Iron Age Thracian culture. The first individual (P192-1) was excavated from a pit sanctuary near Svilengrad, Bulgaria, dated to 800–500 BCE. The other individual (K8) was found in the Yakimova Mogila Tumulus in southeastern Bulgaria, dated to 450–400 BCE. DNA was extracted from teeth using clean room procedures to prevent contamination, following an established protocol [45]. Following the extraction, end-repair and dA-tailing of purified DNA was performed using the Next End Prep Enzyme Mix (New England Biolabs) and following the manufacturer's instructions. Illumina paired-end adapters were ligated, purified and amplified using standard protocols. Ancient DNA in the library preparation was subsequently enriched with WISC, a novel capture protocol using RNA probes transcribed from a modern human DNA library [15]. Pre-capture libraries were then sequenced on the Illumina HiSeq (2×90 bp paired end), whereas post-capture libraries were sequenced on the Illumina MiSeq (2×150 bp paired end).

### Ancient genome data processing

The ancient genome data used for this study come from a number of different sources, which differ both in sample preparation as well as sequencing technology. In order to minimize biases due to these differences as much as possible, we applied very stringent quality control filters during the processing of the different datasets, using the same processing pipeline for each sample when possible. For all samples, pileup files from the aligned reads (reference genome build hg18) were obtained using the ‘mpileup’ command of SAMtools [46], removing PCR duplicates (http://picard.sourceforge.net) as well as low quality alignments (MQ<30) and bases (base quality<30). Due to the higher coverage of the Iceman genome, we performed diploid SNP genotype calling using the Bayesian algorithm implemented in ‘bcftools’ of the SAMtools suite. For all other ancient samples, haploid genotypes were obtained by randomly sampling reads if positions were covered by multiple reads. For all analyses involving only one ancient sample, we excluded DNA damage sites (C>T/G>A transitions) where the ancient sample shows the damage allele (T/A). For analyses including multiple ancient samples, all transitions were excluded.

#### Iceman

Genotype calling was performed using BAM files containing the already aligned reads [5]. Raw genotype calls from bcftools were filtered using ‘vcfutils.pl’ included in SAMtools. The final variants had to pass the following filter criteria in addition to the default filters:

- Phred-scaled variant quality score >40
- Read depth ≥6 or ≤30
- Distance from gaps ≥5 base pairs
- Not within repeat regions (hg18 ‘simpleRepeat’ table, UCSC genome browser)

#### Swedish genomes

Sequencing reads were obtained from the European Nucleotide Archive (ENA), for two samples, Ajv70 (accession ERS084011) and Gok4 (ERS084013). Reads were aligned to the reference genome using bwa [47] with default options.

#### Iberian genomes

The aligned and processed sequencing reads from [14] were filtered according to the criteria described above.

#### Thracian genomes

After merging of overlapping paired end reads (SeqPrep, https://github.com/jstjohn/SeqPrep), the sequencing reads were aligned to the reference genome using bwa with seeding disabled. Pre-and post-capture BAM files from the same sample were then merged and processed as described above. Details on analyses of ancient DNA damage patterns can be found in Table S4 of [15].

#### Archaic hominins

Sequencing reads from the Neanderthal genome project aligned to the human reference genome (hg18) in BAM format were obtained from ftp://ftp.ebi.ac.uk/pub/databases/ensembl/neandertal/BAM_files/Neandertal.bam. BAM files from the low coverage Denisova genome aligned to hg18 were obtained from ftp://hgdownload.cse.ucsc.edu/gbdb/hg18/denisova/SL3003_SL3004_100122-hg18.bam.

### Modern population datasets and processing

For all population genetic analyses, data from the ancient genomes was merged with three different reference datasets of present-day human populations, using only autosomal data. We followed the approach of Skoglund *et al.* [13] and used only variants found in the respective reference dataset, and removing any genotypes with a mismatching alternative allele in the ancient samples.

#### HGPD

Data for 934 unrelated samples from the HGDP from 52 populations, genotyped on the Affymetrix Axiom Human Origins SNP array was obtained from ftp://ftp.cephb.fr/hgdp_supp10/. This dataset was designed for population genetic studies by including 13 different SNP panels with well-defined ascertainment schemes. We used the combined data for all panels (all_snp.* files) as the master dataset for merging with the ancient genomes, after removing SNPs with >10% missing data. For all subsequent analyses, the relevant subset of SNPs corresponding to the desired ascertainment scheme was then extracted from the master dataset. Individuals used for SNP discovery in the ascertainment panels were excluded from all analyses.

#### Complete Genomics genomes

Whole-genome sequence data of 46 samples from 10 populations sequenced to high coverage by Complete Genomics [48] was used for the merge with the ancient samples. Individuals with diploid data were converted to haploid genomes by randomly sampling one allele at all heterozygote genotypes. For the individuals of populations with known recent admixture (African-Americans/ASW; Mexicans/MXL), we created virtual individuals from inferred local admixture tracts for each admixture source populations. For each genome and each source population, we extracted all variants that were homozygous for the respective ancestry and set all other genotypes to missing.

#### 1000 Genomes and Sardinia

The data for this combined dataset were obtained from low-coverage whole-genome sequencing. Therefore, we restricted the analysis to population-level by using only allele frequencies and not individual genotypes. We obtained allele frequency data from 452 unrelated Sardinian individuals sequenced to low coverage as part of the SardiNIA project [49], [50], which was combined with data from the 1000 Genomes project. Data from the phase 1 release, which comprise 1,092 individuals from 14 populations, were obtained from the project ftp site (ftp://ftp.1000genomes.ebi.ac.uk). Population allele frequencies were calculated for each SNP, excluding related individuals, for a final dataset of 841 individuals. Both datasets were merged, excluding variants failing QC filters in any dataset or with mismatching alternative alleles. To further minimize potential biases from the merging of the two datasets, we further removed all variants not passing the more stringent “strict” accessibility mask from the 1000 Genomes project. The remaining variants were converted to hg18 coordinates using the *liftover* tool from the UCSC genome browser (http://genome.ucsc.edu/) for the final merge with the ancient genomes.

### Population structure

The unsupervised maximum-likelihood clustering algorithm implemented in ADMIXTURE [16] was used to cluster each ancient genome with populations of European and Middle Eastern origin in the HGDP dataset. In order circumvent the problem of differing overlap of variants of the ancient samples with HGDP, we first inferred ancestral clusters for the contemporary populations only, and subsequently determined the most likely cluster memberships for each ancient sample using the ancestral allele frequencies of all overlapping. An additional advantage of this strategy is that the genotypes of the ancient samples do not influence the clustering solution for the modern individuals. Initial clustering of the 263 contemporary individuals was performed for *k* = 2 through *k* = 8 ancestral clusters by running ADMIXTURE with default settings. In order to maximize the accuracy for the initial clustering, we used all SNPs where a genotype was observed for any ancient sample prior to removal of damage SNPs. For each value of K, ten replicate runs were performed, and the run with the greatest likelihood was selected for further analysis. The cluster membership proportions for each ancient sample were then obtained by maximizing the log-likelihood function (equation 2 in [16]) using the subset of SNPs with genotype data in the respective ancient sample, and the corresponding ancestral allele frequencies inferred from the modern samples (the P matrix of the ADMIXTURE output). The optimization was implemented using the FRAPPE EM algorithm (equation 4 in [16]). In order to evaluate the accuracy of the inferred clusters for the ancient samples, we repeated the procedure described above for all 263 modern samples and for each subset of SNPs corresponding to the six ancient samples. Cluster assignment accuracy was then determined by calculating the root mean standard error (RMSE) for all combinations of modern population, number of ancestral clusters and ancient sample SNP subset used.

In addition, we also performed principal component analysis (PCA), separately for each ancient genome together with the 263 contemporary individuals, using only non-missing SNPs of the respective ancient sample. The initial PCA was performed using the modern samples only, followed by projection of the ancient samples onto the inferred principal components. All analyses were carried out in R using singular value decomposition (*‘svd’* function) on the genotype matrix.

### Allele sharing and admixture

Patterns of admixture among ancient and modern populations were inferred using a number of related approaches. For the individual-level datasets (CG), we quantified the amount of derived allele sharing between the ancient and modern genomes. For each ancient genome, we extracted all variants showing the derived allele and calculated the proportion of variants with matching alleles for each of the modern genomes. The sharing rate was normalized by the rate for the individual with the lowest sharing rate with the Iceman (NA18508, YRI), for easier comparison among different ancient genomes.

For population-level datasets (1000G/Sardinia), we used *TreeMix* [19] to infer a maximum-likelihood population tree and putative admixture events. The program was run separately for each ancient genome together with the contemporary populations, using only SNPs without missing data in the ancient sample. For the HGDP dataset, only the Iceman was used due to the low number of SNPs for the other ancient samples. The maximum number of migration edges added was 5, and we selected the run with the maximum likelihood from 10 replicate runs for each migration edge parameter. The number of SNPs used for the LD correction was adjusted to the total number of SNPs for each ancient sample (Table S6).

Additionally, we used formal tests of admixture as implemented in the three- and four-population tests [17], [18] for all datasets. Standard errors for the statistics were determined using a weighted block jackknife, with a window size of 5 Mb.

### Estimation of divergence times

Divergence times of the Iceman with contemporary genomes was estimated using a previously described coalescent method [13], [21], based on the number of concordant and discordant genealogical topologies. For the CG dataset, divergence time was calculated in turn for each genome, using the two gene copies of the modern genome at each SNP to determine concordant/discordant status. Heterozygous sites were randomly assigned to the two resulting haploid genomes. For the 1000G/Sardinia dataset no individual-level genotypes were available, so we used a slightly modified approach. For each modern population, two virtual haploid genomes were generated by randomly sampling alleles with probability equal to their derived allele frequency at each SNP, and divergence time was estimated as above. Under the assumption of panmixia in each population, the resulting times can be interpreted as the divergence of an average individual from that population to the Iceman. The resulting coalescent times were converted to time in years using population-specific effective population sizes for African, European and Asian population from Gronau *et al.* [23] (Africa: 29,300 (23,900–35,200); Europe: 5,800 (900–11,500); Asia: 3,000 (300–6,600)), assuming a generation time of 25 years. For the lower and upper bounds of the converted divergence time we combined the uncertainty of both our divergence estimates and the effective population size estimated by Gronau *et al.* [23].

## Supporting Information

Figure S1 ADMIXTURE results for HGDP. Panels show the results for ADMIXTURE runs for *k* = 2 to *k* = 8 ancestral clusters on the HGDP individuals, and the corresponding cluster proportions inferred for the ancient samples.
(PDF)

Figure S2 PCA results for HGDP. Panels show the results for PCA on the HGDP individuals for subsets of SNPs with data in the respective ancient sample. Each point represents an individual, with plot symbol and color indicating population of origin. The position of the ancient samples was inferred by projecting onto the PC space calculated using the modern samples only.
(PDF)

Figure S3 Derived allele sharing with CG genomes. Normalized derived allele sharing rate of the Iceman with the full set of whole genomes from Complete Genomics. Each circle represents the rate of sharing with a particular genome, grouped by population of origin (circle color). Positions on the y-axis have added jitter for ease of visualization. Population names with suffix correspond to the respective ancestry tracts (EUR: European; YRI: African; NAH: Native American) of individuals of populations with known recent admixture (ASW, MXL).
(PDF)

Figure S4 D-tests of Iceman with European populations from CG. Each diamond indicates the value of the D-statistic for a single D-test involving the Iceman and a pair of modern European populations, indicated on the y-axis. A significant deviation from the x = 0 line indicates a closer relationship of the ancient sample to one of the populations in the tested pair. Significance at Z = 3 is indicated with black diamonds, and the line shows the corresponding standard error of the D-statistic. Populations with EUR suffix correspond to the European ancestry tracts of individuals of populations with known European admixture (ASW, MXL).
(PDF)

Figure S5 D-tests of ancient samples with European populations from 1000G/Sardinia. In each panel, diamonds indicate the value of the D-statistic for a single D-test involving the respective ancient samples and a pair of modern European populations. Significance at Z = 3 is indicated with filled diamonds, and the line shows the corresponding standard error of the D-statistic. Plot colors indicate different pairs of geographic regions within Europe.
(PDF)

Figure S6 Results of TreeMix analysis of the Iceman with 1000G/Sardinia. Shown are maximum-likelihood trees and the matrices of pairwise residuals for all models allowing from *m* = 0 to *m* = 5 mixture events.
(PDF)

Figure S7 RMSE for HGDP cluster proportions. Panels represent the results for ancestral cluster membership accuracy for each subset of SNPs corresponding to the ancient samples. Accuracy is measured as root mean square error (RMSE) of cluster membership proportions for each population of origin and value of *k*.
(PDF)

Figure S8 Results of TreeMix analysis of the ancient samples with 1000G/Sardinia. Shown are maximum-likelihood trees and the matrices of pairwise residuals for all ancient samples, without mixture edges. Bootstrap support for the position of the ancient sample is indicated by the numbers next to the branches.
(PDF)

Figure S9 PCA with archaic hominins and Iceman in HGDP. Plot symbols indicate the position of an individual, projected onto PC space inferred using Chimp, Neanderthal and Denisova. Individuals are colored according to continental region of origin.
(PDF)

Figure S10 D-tests of archaic hominins with CG populations and Iceman. In each panel, diamonds indicate the value of the D-statistic for a single D-test involving the respective archaic hominin sample and pairs of Iceman/CG population on the y-axis. A significant shift to the left (D<0) indicates a closer relationship of the ancient sample to the Iceman compared to the modern population. Significance at Z = 3 is indicated with filled diamonds, and the line shows the corresponding standard error of the D-statistic. Plot colors indicate geographic origin of the CG population.
(PDF)

Table S1 D-Tests of Iceman – 1000G/Sardinia with outgroup.
(XLSX)

Table S2 Divergence time estimates of the Iceman with Complete Genomics genomes.
(XLSX)

Table S3 Divergence time estimates of the Iceman with 1000G/Sardinia.
(XLSX)

Table S4 Signals of admixture using the *f_3_* statistic with modern populations.
(XLSX)

Table S5 Signals of admixture using the *f_3_* statistic with ancient samples.
(XLSX)

Table S6 TreeMix parameters.
(XLSX)

## References

[1] Seidler H, Bernhard W, Teschler-Nicola M, Platzer W, zur Nedden D, et al (1992) Some anthropological aspects of the prehistoric Tyrolean ice man. Science 258: 455–457. 1411539 10.1126/science.1411539

[2] Murphy WA, zur Nedden D, Gostner P, Knapp R, Recheis W, et al (2003) The Iceman: Discovery and Imaging. Radiology 226: 614–629. 12601185 10.1148/radiol.2263020338

[3] Kutschera W, Golser R, Priller A, Rom W, Steier P, et al.. (2000) Radiocarbon dating of equipment from the Iceman. In: Bortenschlager U-PMDS, Oeggl U-PDK, editors. The Iceman and his Natural Environment: Palaeobotanical Results (The Man in the Ice). Vienna: Springer. pp. 1–9.

[4] Rollo F, Ubaldi M, Ermini L, Marota I (2002) Ötzi's last meals: DNA analysis of the intestinal content of the Neolithic glacier mummy from the Alps. Proc Natl Acad Sci U S A 99: 12594–12599. 12244211 10.1073/pnas.192184599 PMC130505

[5] Keller A, Graefen A, Ball M, Matzas M, Boisguerin V, et al (2012) New insights into the Tyrolean Iceman's origin and phenotype as inferred by whole-genome sequencing. Nature Communications 3: 698. 10.1038/ncomms1701 22426219

[6] Müller W, Fricke H, Halliday AN, McCulloch MT, Wartho J-A (2003) Origin and Migration of the Alpine Iceman. Science 302: 862–866. 14593178 10.1126/science.1089837

[7] Bramanti B, Thomas MG, Haak W, Unterlaender M, Jores P, et al (2009) Genetic Discontinuity Between Local Hunter-Gatherers and Central Europe's First Farmers. Science 326: 137–140. 19729620 10.1126/science.1176869

[8] Haak W, Balanovsky O, Sanchez JJ, Koshel S, Zaporozhchenko V, et al (2010) Ancient DNA from European Early Neolithic Farmers Reveals Their Near Eastern Affinities. PLoS Biol 8: e1000536. 21085689 10.1371/journal.pbio.1000536 PMC2976717

[9] Lacan M, Keyser C, Ricaut F-X, Brucato N, Duranthon F, et al (2011) Ancient DNA reveals male diffusion through the Neolithic Mediterranean route. Proc Natl Acad Sci U S A 108: 9788–9791. 21628562 10.1073/pnas.1100723108 PMC3116412

[10] Der Sarkissian C, Balanovsky O, Brandt G, Khartanovich V, Buzhilova A, et al (2013) Ancient DNA Reveals Prehistoric Gene-Flow from Siberia in the Complex Human Population History of North East Europe. PLoS Genet 9: e1003296. 23459685 10.1371/journal.pgen.1003296 PMC3573127

[11] Brotherton P, Haak W, Templeton J, Brandt G, Soubrier J, et al (2013) Neolithic mitochondrial haplogroup H genomes and the genetic origins of Europeans. Nat Commun 4: 1764. 23612305 10.1038/ncomms2656 PMC3978205

[12] Fu Q, Rudan P, Pääbo S, Krause J (2012) Complete Mitochondrial Genomes Reveal Neolithic Expansion into Europe. PLoS ONE 7: e32473. 22427842 10.1371/journal.pone.0032473 PMC3302788

[13] Skoglund P, Malmström H, Raghavan M, Storå J, Hall P, et al (2012) Origins and Genetic Legacy of Neolithic Farmers and Hunter-Gatherers in Europe. Science 336: 466–469. 22539720 10.1126/science.1216304

[14] Sánchez-Quinto F, Schroeder H, Ramirez O, Ávila-Arcos MC, Pybus M, et al (2012) Genomic Affinities of Two 7,000-Year-Old Iberian Hunter-Gatherers. Current Biology 22: 1494–1499. 22748318 10.1016/j.cub.2012.06.005

[15] Carpenter ML, Buenrostro JD, Valdiosera C, Schroeder H, Allentoft ME, et al (2013) Pulling out the 1%: Whole-Genome Capture for the Targeted Enrichment of Ancient DNA Sequencing Libraries. The American Journal of Human Genetics 93: 852–864. 24568772 10.1016/j.ajhg.2013.10.002 PMC3824117

[16] Alexander DH, Novembre J, Lange K (2009) Fast model-based estimation of ancestry in unrelated individuals. Genome Res 19: 1655–1664. 19648217 10.1101/gr.094052.109 PMC2752134

[17] Durand EY, Patterson N, Reich D, Slatkin M (2011) Testing for Ancient Admixture between Closely Related Populations. Molecular Biology and Evolution 28: 2239–2252. 21325092 10.1093/molbev/msr048 PMC3144383

[18] Patterson N, Moorjani P, Luo Y, Mallick S, Rohland N, et al (2012) Ancient Admixture in Human History. Genetics 192: 1065–1093. 22960212 10.1534/genetics.112.145037 PMC3522152

[19] Pickrell JK, Pritchard JK (2012) Inference of Population Splits and Mixtures from Genome-Wide Allele Frequency Data. PLoS Genet 8: e1002967. 23166502 10.1371/journal.pgen.1002967 PMC3499260

[20] Fu Q, Meyer M, Gao X, Stenzel U, Burbano HA, et al (2013) DNA analysis of an early modern human from Tianyuan Cave, China. Proc Natl Acad Sci U S A 110: 2223–2227. 23341637 10.1073/pnas.1221359110 PMC3568306

[21] Skoglund P, Götherström A, Jakobsson M (2011) Estimation of Population Divergence Times from Non-Overlapping Genomic Sequences: Examples from Dogs and Wolves. Mol Biol Evol 28: 1505–1517. 21177316 10.1093/molbev/msq342

[22] Gravel S, Henn BM, Gutenkunst RN, Indap AR, Marth GT, et al (2011) Demographic history and rare allele sharing among human populations. Proc Natl Acad Sci U S A 108: 11983–11988. 21730125 10.1073/pnas.1019276108 PMC3142009

[23] Gronau I, Hubisz MJ, Gulko B, Danko CG, Siepel A (2011) Bayesian inference of ancient human demography from individual genome sequences. Nat Genet 43: 1031–1034. 21926973 10.1038/ng.937 PMC3245873

[24] Ralph P, Coop G (2013) The Geography of Recent Genetic Ancestry across Europe. PLoS Biol 11: e1001555. 23667324 10.1371/journal.pbio.1001555 PMC3646727

[25] Botigué LR, Henn BM, Gravel S, Maples BK, Gignoux CR, et al (2013) Gene flow from North Africa contributes to differential human genetic diversity in southern Europe. Proc Natl Acad Sci U S A 110: 11791–11796. 23733930 10.1073/pnas.1306223110 PMC3718088

[26] Moorjani P, Patterson N, Hirschhorn JN, Keinan A, Hao L, et al (2011) The History of African Gene Flow into Southern Europeans, Levantines, and Jews. PLoS Genet 7: e1001373. 21533020 10.1371/journal.pgen.1001373 PMC3080861

[27] Derenko M, Malyarchuk B, Denisova G, Wozniak M, Grzybowski T, et al (2007) Y-chromosome haplogroup N dispersals from south Siberia to Europe. J Hum Genet 52: 763–770. 17703276 10.1007/s10038-007-0179-5

[28] Salmela E, Lappalainen T, Fransson I, Andersen PM, Dahlman-Wright K, et al (2008) Genome-Wide Analysis of Single Nucleotide Polymorphisms Uncovers Population Structure in Northern Europe. PLoS ONE 3: e3519. 18949038 10.1371/journal.pone.0003519 PMC2567036

[29] The International HapMap 3 Consortium (2010) Integrating common and rare genetic variation in diverse human populations. Nature 467: 52–58. 20811451 10.1038/nature09298 PMC3173859

[30] Henn BM, Gignoux CR, Jobin M, Granka JM, Macpherson JM, et al (2011) Hunter-gatherer genomic diversity suggests a southern African origin for modern humans. Proc Natl Acad Sci U S A 108: 5154–5162. 21383195 10.1073/pnas.1017511108 PMC3069156

[31] Wall JD, Yang MA, Jay F, Kim SK, Durand EY, et al (2013) Higher Levels of Neanderthal Ancestry in East Asians than in Europeans. Genetics 194: 199–209. 23410836 10.1534/genetics.112.148213 PMC3632468

[32] Novembre J, Johnson T, Bryc K, Kutalik Z, Boyko AR, et al (2008) Genes mirror geography within Europe. Nature 456: 98–101. 18758442 10.1038/nature07331 PMC2735096

[33] Lao O, Lu TT, Nothnagel M, Junge O, Freitag-Wolf S, et al (2008) Correlation between Genetic and Geographic Structure in Europe. Current Biology 18: 1241–1248. 18691889 10.1016/j.cub.2008.07.049

[34] Jay F, Sjödin P, Jakobsson M, Blum MGB (2013) Anisotropic Isolation by Distance: The Main Orientations of Human Genetic Differentiation. Mol Biol Evol 30: 513–525. 23171862 10.1093/molbev/mss259 PMC3563970

[35] Reich D, Green RE, Kircher M, Krause J, Patterson N, et al (2010) Genetic history of an archaic hominin group from Denisova Cave in Siberia. Nature 468: 1053–1060. 21179161 10.1038/nature09710 PMC4306417

[36] Green RE, Krause J, Briggs AW, Maricic T, Stenzel U, et al (2010) A Draft Sequence of the Neandertal Genome. Science 328: 710–722. 20448178 10.1126/science.1188021 PMC5100745

[37] Cavalli-Sforza LL, Piazza A (1993) Human genomic diversity in Europe: a summary of recent research and prospects for the future. Eur J Hum Genet 1: 3–18. 7520820 10.1159/000472383

[38] D'Amore G, Di Marco S, Floris G, Pacciani E, Sanna E (2010) Craniofacial morphometric variation and the biological history of the peopling of Sardinia. HOMO - Journal of Comparative Human Biology 61: 385–412. 20979998 10.1016/j.jchb.2010.09.002

[39] Caramelli D, Vernesi C, Sanna S, Sampietro L, Lari M, et al (2007) Genetic variation in prehistoric Sardinia. Hum Genet 122: 327–336. 17629747 10.1007/s00439-007-0403-6

[40] Ghirotto S, Mona S, Benazzo A, Paparazzo F, Caramelli D, et al (2010) Inferring Genealogical Processes from Patterns of Bronze-Age and Modern DNA Variation in Sardinia. Mol Biol Evol 27: 875–886. 19955482 10.1093/molbev/msp292

[41] Brandt G, Haak W, Adler CJ, Roth C, Szécsényi-Nagy A, et al (2013) Ancient DNA Reveals Key Stages in the Formation of Central European Mitochondrial Genetic Diversity. Science 342: 257–261. 24115443 10.1126/science.1241844 PMC4039305

[42] Pinhasi R, Thomas MG, Hofreiter M, Currat M, Burger J (2012) The genetic history of Europeans. Trends in Genetics 28: 496–505. 22889475 10.1016/j.tig.2012.06.006

[43] Lazaridis I, Patterson N, Mittnik A, Renaud G, Mallick S, et al. (2013) Ancient human genomes suggest three ancestral populations for present-day Europeans. bioRxiv. Available: http://biorxiv.org/content/early/2013/12/23/001552. Accesed 14 April 2014. 10.1038/nature13673 PMC4170574 25230663

[44] Rasmussen M, Guo X, Wang Y, Lohmueller KE, Rasmussen S, et al (2011) An Aboriginal Australian Genome Reveals Separate Human Dispersals into Asia. Science 334: 94–98. 21940856 10.1126/science.1211177 PMC3991479

[45] Yang DY, Eng B, Waye JS, Dudar JC, Saunders SR (1998) Improved DNA extraction from ancient bones using silica-based spin columns. American Journal of Physical Anthropology 105: 539–543. 9584894 10.1002/(SICI)1096-8644(199804)105:4<539::AID-AJPA10>3.0.CO;2-1

[46] Li H, Handsaker B, Wysoker A, Fennell T, Ruan J, et al (2009) The Sequence Alignment/Map format and SAMtools. Bioinformatics 25: 2078–2079. 19505943 10.1093/bioinformatics/btp352 PMC2723002

[47] Li H, Durbin R (2009) Fast and accurate short read alignment with Burrows–Wheeler transform. Bioinformatics 25: 1754–1760. 19451168 10.1093/bioinformatics/btp324 PMC2705234

[48] Kidd JM, Gravel S, Byrnes J, Moreno-Estrada A, Musharoff S, et al (2012) Population Genetic Inference from Personal Genome Data: Impact of Ancestry and Admixture on Human Genomic Variation. The American Journal of Human Genetics 91: 660–671. 23040495 10.1016/j.ajhg.2012.08.025 PMC3484644

[49] Francalacci P, Morelli L, Angius A, Berutti R, Reinier F, et al (2013) Low-Pass DNA Sequencing of 1200 Sardinians Reconstructs European Y-Chromosome Phylogeny. Science 341: 565–569. 23908240 10.1126/science.1237947 PMC5500864

[50] Orrù V, Steri M, Sole G, Sidore C, Virdis F, et al (2013) Genetic Variants Regulating Immune Cell Levels in Health and Disease. Cell 155: 242–256. 24074872 10.1016/j.cell.2013.08.041 PMC5541764

