# Supplementary figures and images for: Population Genomic Analysis of Ancient and Modern Genomes Yields New Insights into the Genetic Ancestry of the Tyrolean Iceman and the Genetic Structure of Europe
Source: PLoS Genet. 2014 May 8;10(5):e1004353. doi: 10.1371/journal.pgen.1004353 (PMC4014435; doi:10.1371/journal.pgen.1004353)

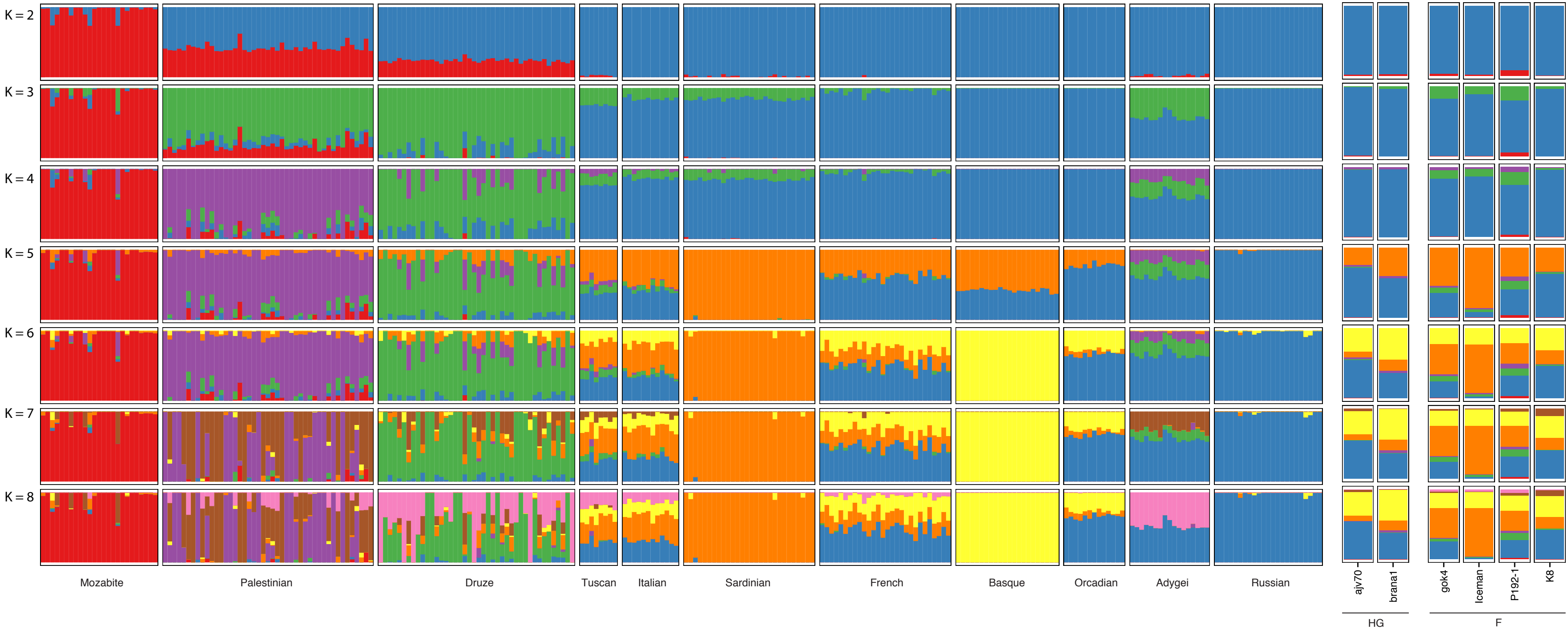

Supplement: Figure S1 — ADMIXTURE results for HGDP. Panels show the results for ADMIXTURE runs for k = 2 to k = 8 ancestral clusters on the HGDP individuals, and the corresponding cluster proportions inferred for the ancient samples. (PDF) [file pgen.1004353.s001.pdf]

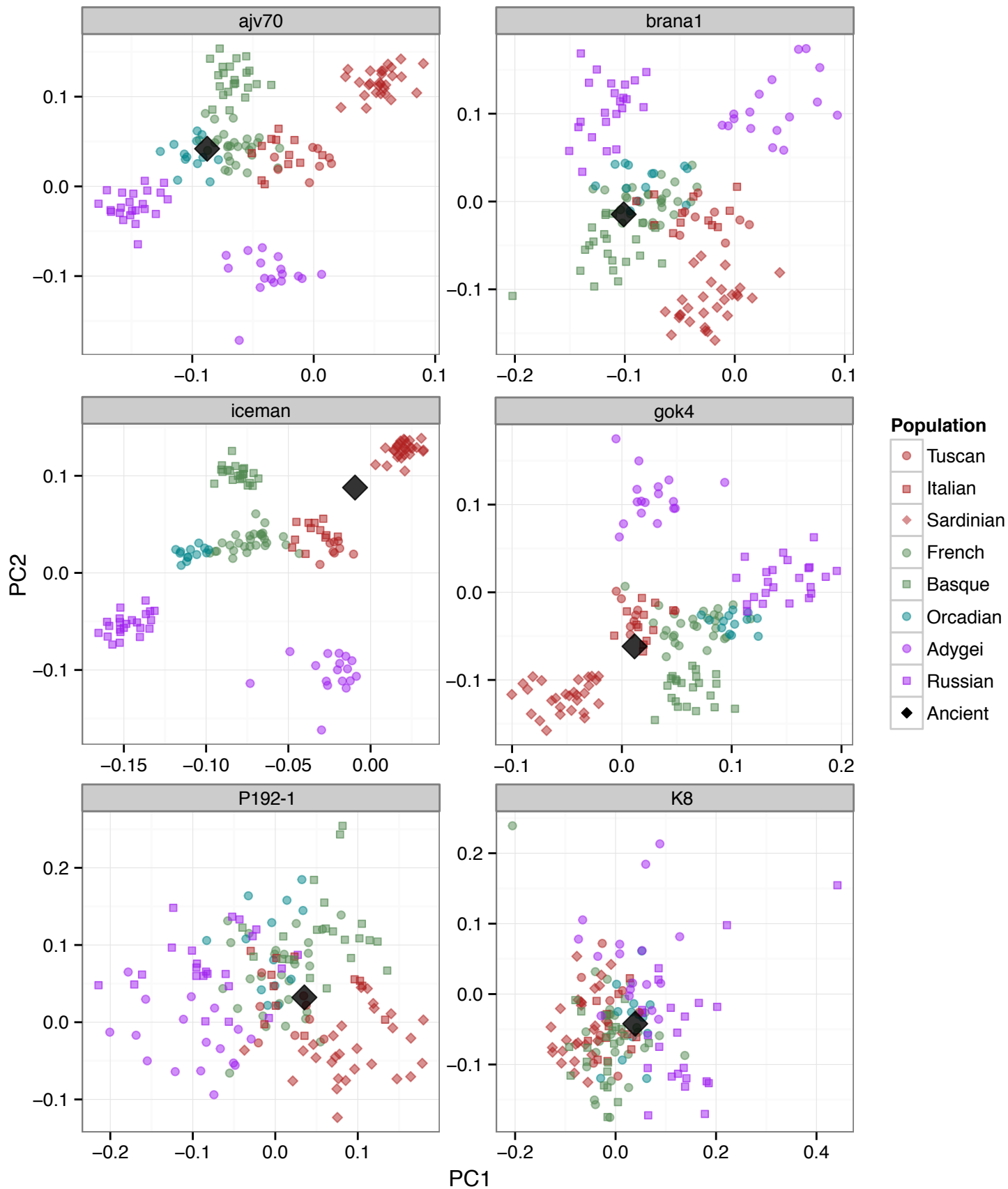

Supplement: Figure S2 — PCA results for HGDP. Panels show the results for PCA on the HGDP individuals for subsets of SNPs with data in the respective ancient sample. Each point represents an individual, with plot symbol and color indicating population of origin. The position of the ancient samples was inferred by projecting onto the PC space calculated using the modern samples only. (PDF) [file pgen.1004353.s002.pdf]

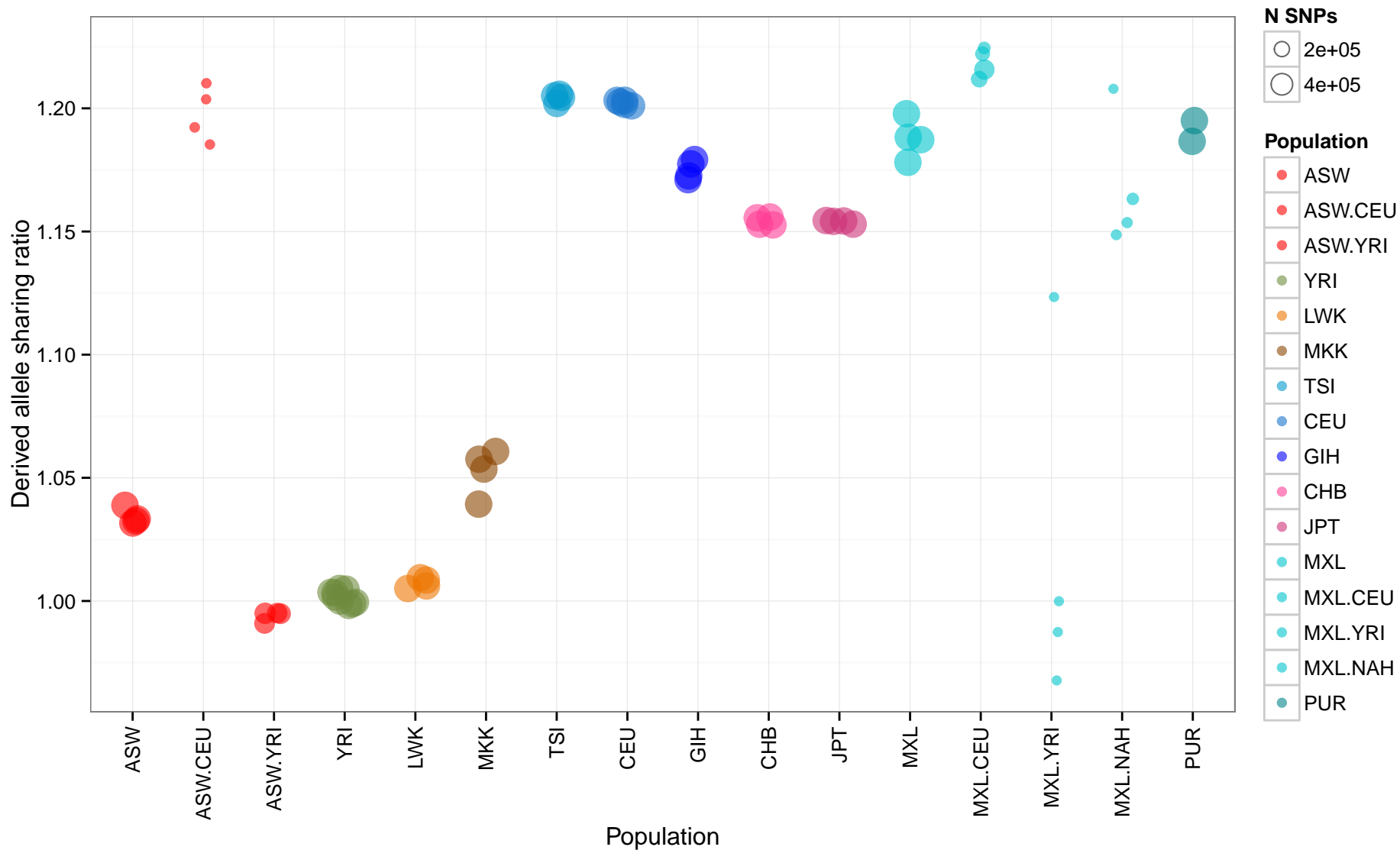

Supplement: Figure S3 — Derived allele sharing with CG genomes. Normalized derived allele sharing rate of the Iceman with the full set of whole genomes from Complete Genomics. Each circle represents the rate of sharing with a particular genome, grouped by population of origin (circle color). Positions on the y-axis have added jitter for ease of visualization. Population names with suffix correspond to the respective ancestry tracts (EUR: European; YRI: African; NAH: Native American) of individuals of populations with known recent admixture (ASW, MXL). (PDF) [file pgen.1004353.s003.pdf]

Configuration

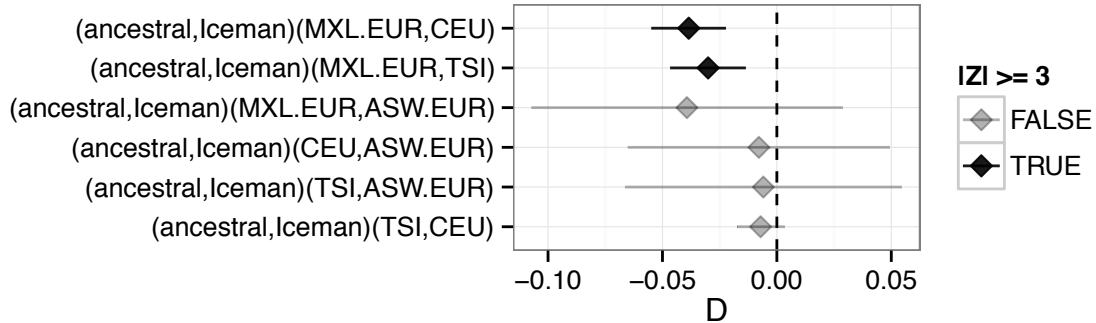

Supplement: Figure S4 — D-tests of Iceman with European populations from CG. Each diamond indicates the value of the D-statistic for a single D-test involving the Iceman and a pair of modern European populations, indicated on the y-axis. A significant deviation from the x = 0 line indicates a closer relationship of the ancient sample to one of the populations in the tested pair. Significance at Z = 3 is indicated with black diamonds, and the line shows the corresponding standard error of the D-statistic. Populations with EUR suffix correspond to the European ancestry tracts of individuals of populations with known European admixture (ASW, MXL). (PDF) [file pgen.1004353.s004.pdf]

Configuration

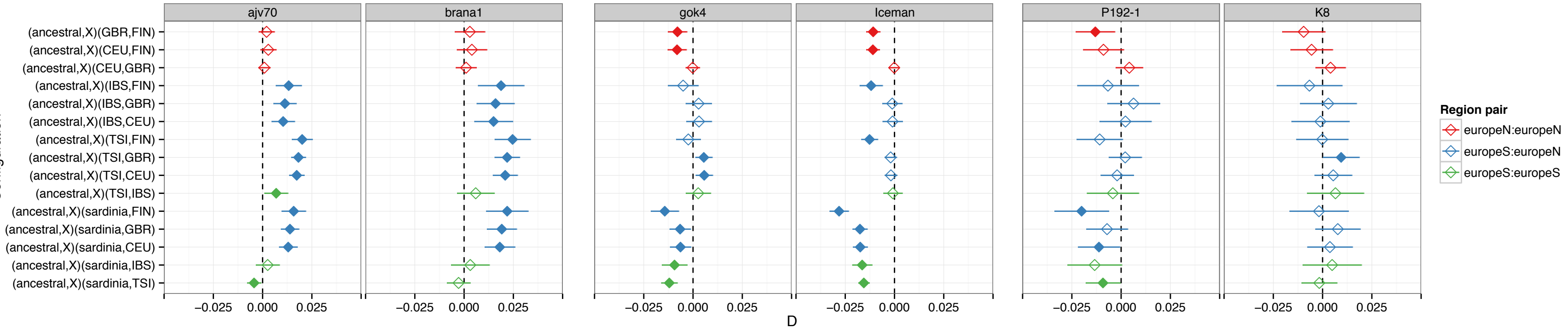

Supplement: Figure S5 — D-tests of ancient samples with European populations from 1000G/Sardinia. In each panel, diamonds indicate the value of the D-statistic for a single D-test involving the respective ancient samples and a pair of modern European populations. Significance at Z = 3 is indicated with filled diamonds, and the line shows the corresponding standard error of the D-statistic. Plot colors indicate different pairs of geographic regions within Europe. (PDF) [file pgen.1004353.s005.pdf]

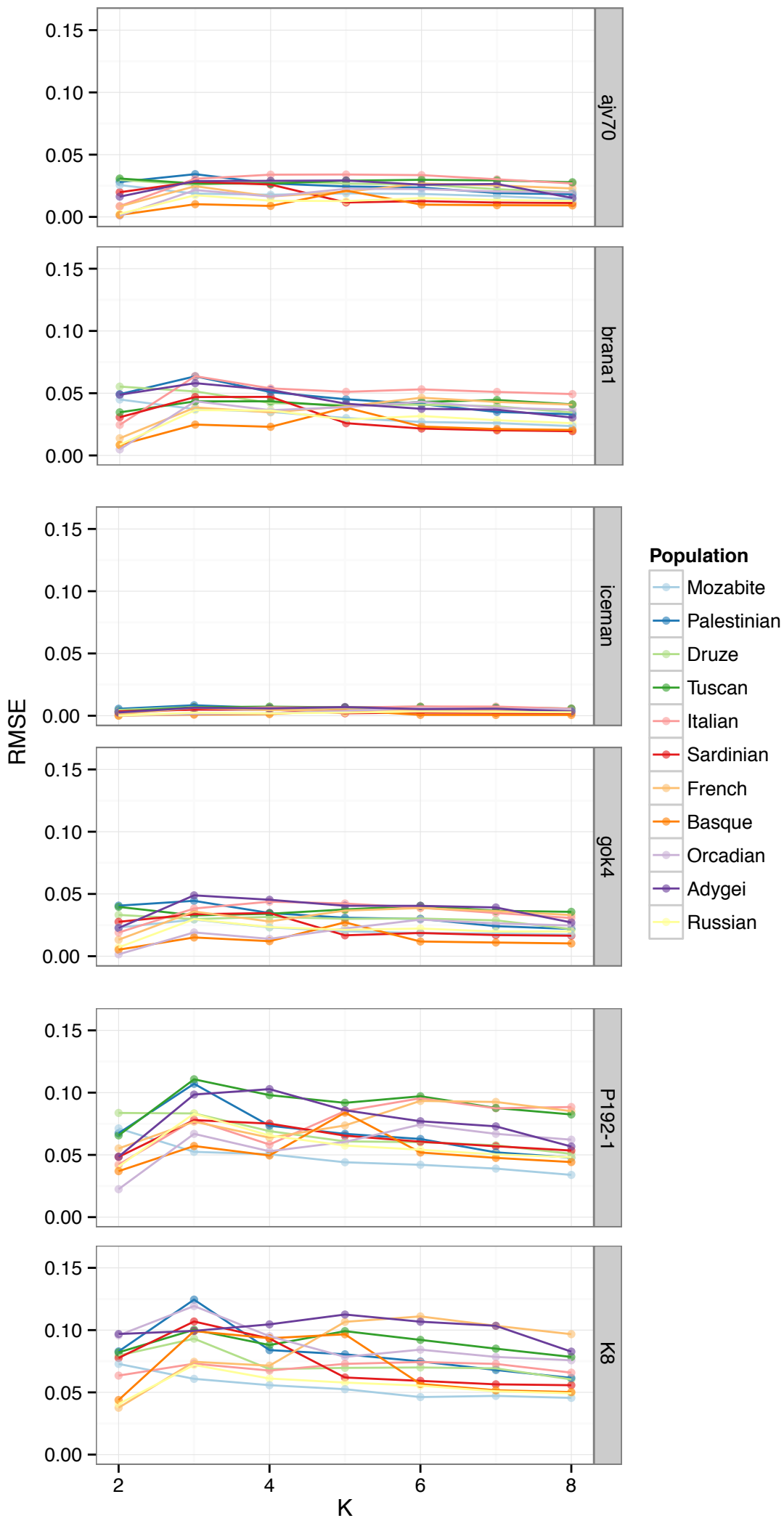

Supplement: Figure S7 — RMSE for HGDP cluster proportions. Panels represent the results for ancestral cluster membership accuracy for each subset of SNPs corresponding to the ancient samples. Accuracy is measured as root mean square error (RMSE) of cluster membership proportions for each population of origin and value of k. (PDF) [file pgen.1004353.s007.pdf]

ajv70

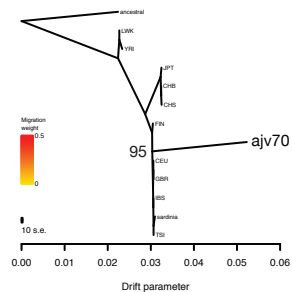

brana1

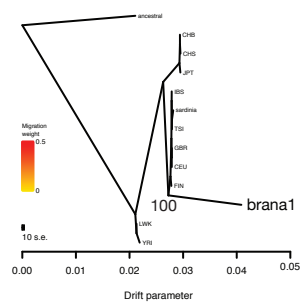

gok4

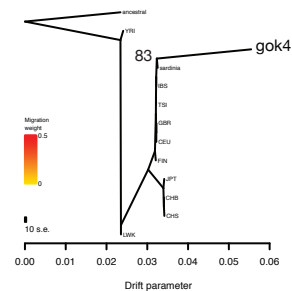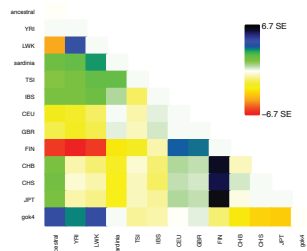

P192-1

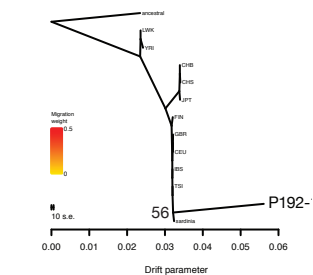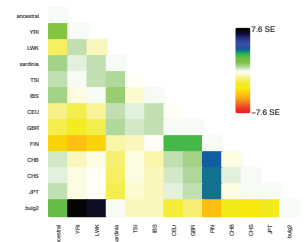

K8

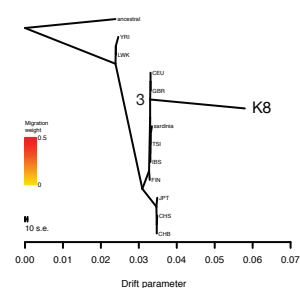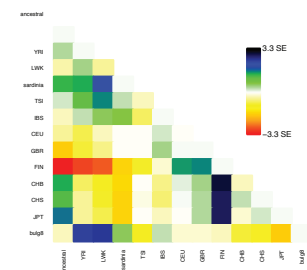

Supplement: Figure S8 — Results of TreeMix analysis of the ancient samples with 1000G/Sardinia. Shown are maximum-likelihood trees and the matrices of pairwise residuals for all ancient samples, without mixture edges. Bootstrap support for the position of the ancient sample is indicated by the numbers next to the branches. (PDF) [file pgen.1004353.s008.pdf]

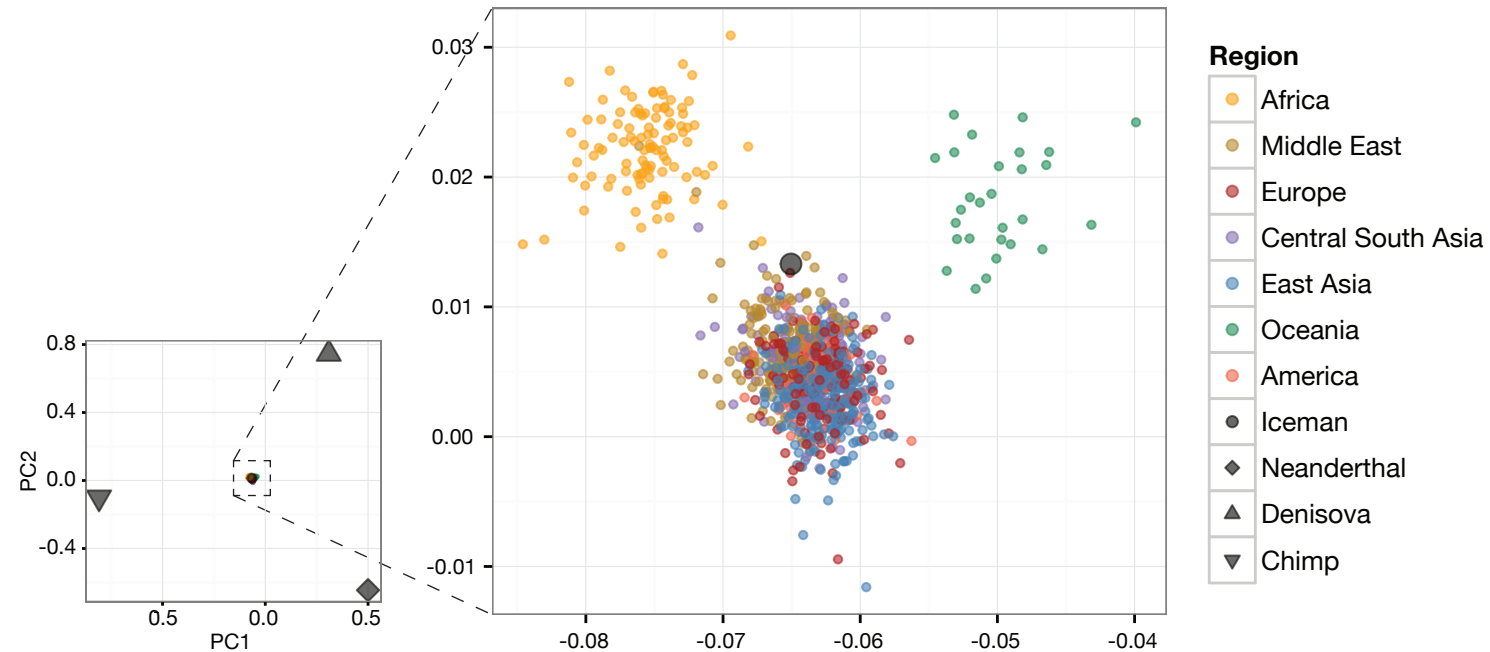

Supplement: Figure S9 — PCA with archaic hominins and Iceman in HGDP. Plot symbols indicate the position of an individual, projected onto PC space inferred using Chimp, Neanderthal and Denisova. Individuals are colored according to continental region of origin. (PDF) [file pgen.1004353.s009.pdf]

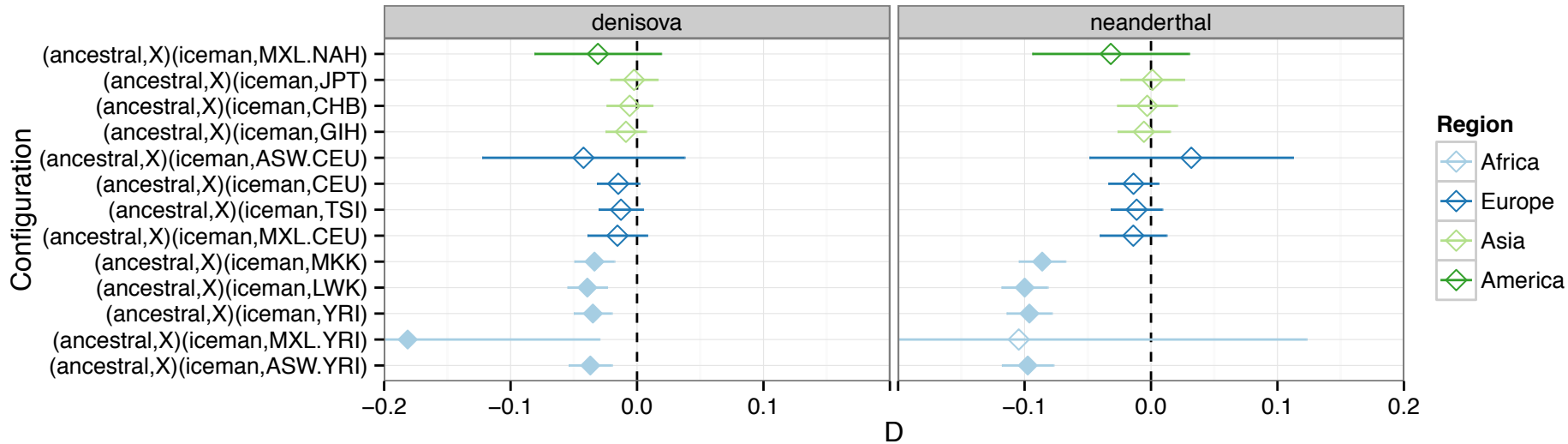

Supplement: Figure S10 — D-tests of archaic hominins with CG populations and Iceman. In each panel, diamonds indicate the value of the D-statistic for a single D-test involving the respective archaic hominin sample and pairs of Iceman/CG population on the y-axis. A significant shift to the left (D<0) indicates a closer relationship of the ancient sample to the Iceman compared to the modern population. Significance at Z = 3 is indicated with filled diamonds, and the line shows the corresponding standard error of the D-statistic. Plot colors indicate geographic origin of the CG population. (PDF) [file pgen.1004353.s010.pdf]
