# Supplementary material for: Population Genomic Analysis of Ancient and Modern Genomes Yields New Insights into the Genetic Ancestry of the Tyrolean Iceman and the Genetic Structure of Europe
Source: PLoS Genet. 2014 May 8;10(5):e1004353. doi: 10.1371/journal.pgen.1004353 (PMC4014435; doi:10.1371/journal.pgen.1004353)
Supplement: Figure S6 — Results of TreeMix analysis of the Iceman with 1000G/Sardinia. Shown are maximum-likelihood trees and the matrices of pairwise residuals for all models allowing from m = 0 to m = 5 mixture events. (PDF) [file pgen.1004353.s006.pdf]

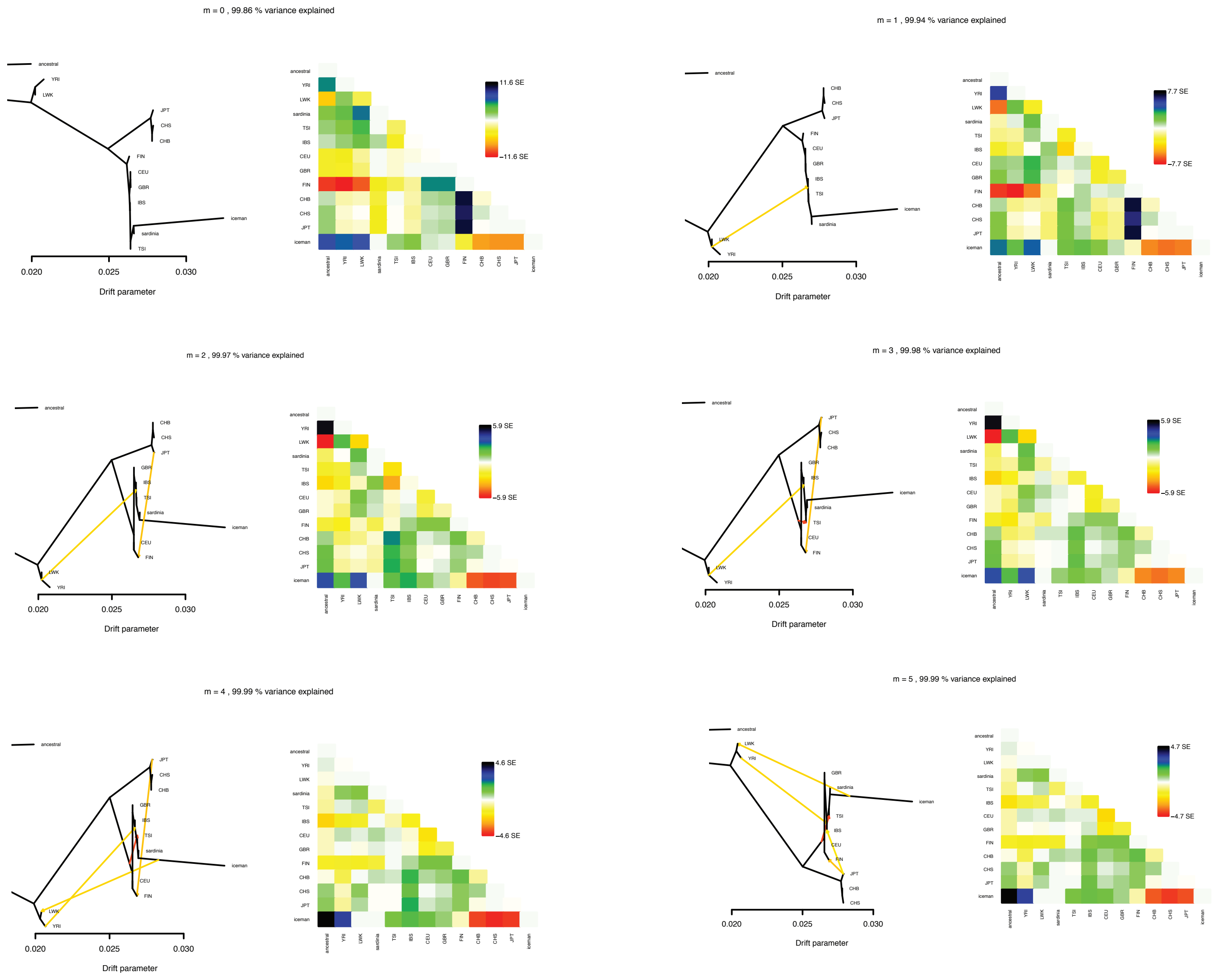
m = 0 , 99.86 % variance explained
11.6 SE
−11.6 SE
0.020
0.025
0.030
Drift parameter
m = 1 , 99.94 % variance explained
7.7 SE
−7.7 SE
0.020
0.025
0.030
Drift parameter
m = 2 , 99.97 % variance explained
5.9 SE
−5.9 SE
0.020
0.025
0.030
Drift parameter
m = 3 , 99.98 % variance explained
5.9 SE
−5.9 SE
0.020
0.025
0.030
Drift parameter
m = 4 , 99.99 % variance explained
4.6 SE
−4.6 SE
0.020
0.025
0.030
Drift parameter
m = 5 , 99.99 % variance explained
4.7 SE
−4.7 SE
0.020
0.025
0.030
Drift parameter
ancestral
YRI
LWK
sardinia
TSI
IBS
CEU
GBR
FIN
CHB
CHS
JPT
iceman
